# EP13 A case of candida albicans septic sacroiliitis complicating severe COVID-19 infection

**DOI:** 10.1093/rap/rkaa052.012

**Published:** 2020-11-03

**Authors:** Ioana Onac, Saadia Ali, Arti Mahto, Andrew Rutherford, James Galloway, Deepak Nagra

**Affiliations:** King's College Hospital, London, United Kingdom

## Abstract

**Case report - Introduction:**

Bacterial and fungal infections are recognised complications of viral pneumonia, particularly in patients who are critically ill. We describe a case of fungal sacroiliitis complicating severe COVID-19 pneumonia following a prolonged intensive care unit (ICU) admission.

Candida albicans sacroilitis is a rarely reported infection with few case reports in the literature. Candida osteoarticular infections can present as septic arthritis, with knee involvement in 75% of cases, or osteomyelitis. The latter presentation differs based on age - vertebral involvement (51%) is more common in adults while children are more likely to present with infection in the long bones, ribs, or sternum.

**Case report - Case description:**

A 48-year-old Afro-Caribbean gentleman with a history of hypertension and obesity was admitted to the ICU with clinical, laboratory and radiographic features of COVID-19 infection despite persistently negative swabs. Whilst in ICU he required mechanical ventilation. His stay was further complicated by multiple infections, pulmonary emboli, and the presence of a cavitating lesion in the left lung. Cultures from bronchoalveolar lavage and a central venous catheter line grew Serratia Mascense, candida glabrata and pseudomonas were isolated from his urine. He was treated with multiple antibiotics including meropenem, tazocin, ceftazidime and avibactam.

After 61 days in the ICU he was transferred to the ward. He developed severe pain in his right hip which was worse on movement. This was followed by urinary incontinence and sensory deficit in the right L2/L3 dermatome. He underwent magnetic resonance imaging (MRI) of his spine and sacroiliac joints which showed right sided sacroiliitis and oedema around the iliopsoas muscle. He was started on vancomycin, later changed to ceftazidime avibactam and metronidazole. An echocardiogram did not show any vegetations. He underwent a biopsy of his sacroiliac joints which confirmed the presence of leucocytes, extended cultures yielded candida albicans in one out of two biopsy specimens.

Considering ongoing pyrexia, pain and inflammatory markers, intravenous fluconazole was added to his antibiotic regimen which resulted in a marked improvement in mobility. After four weeks, ceftazidime, metronidazole and avibactam were stopped, and fluconazole was administered as oral tablets. 6 days later he became febrile and IV fluconazole was restarted.

A repeat chest CT showed resolution of the cavity but ongoing changes suggestive of organising pneumonia. A repeat MRI of the sacroiliac joints revealed minor improvement. Intravenous Fluconazole was continued for a total of 8 weeks and was changed to tablets for complete a total of 12 weeks.

**Case report - Discussion:**

This is a severe case of COVID-19 infection who despite 9 negative PCR tests, on day 53, had positive IgG for SARS-CoV-2 infection, confirming our clinical suspicion. Particularly in the ICU setting, individuals are approximately ten times more likely to have secondary bacterial/fungal infections with more frequent detection of multidrug-resistant Gram- negative pathogens.

This case highlights several difficulties. Urine cultures had confirmed candida albicans, likely to be related to catheter related urinary tract infections, and a possible source for our patient but also a resistant pseudomonas aeruginosa species. Furthermore, cultures were positive for Serratia Mascense, candida glabrata. He had also already been treated with prolonged, broad spectrum antimicrobial treatment. Considering this, establishing the aetiology of the septic sacroiliitis was challenging. The rarity of candida sacroiliitis and presence of the organism in just one specimen made this more difficult. This led to the decision of a repeat sacroiliac biopsy to supply sufficient samples for further microbial analyses such as 16S, 18S and mycobacteria culture, all of which were negative.

He became febrile after the discontinuation of antimicrobials and a switch to oral fluconazole therapy. He was extensively re-investigated and despite resolution of the lung cavity, there were changes which could have been consistent with an organising pneumonia. At this point he was neutropenic, mildly eosinophilic, and therefore a drug reaction was also considered.

Repeat MRI revealed resolving muscle inflammation and minimal change at the bone site, with erosions and possible reactive bone marrow oedema. Following discussion with microbiology the decision was made to persist with intravenous Fluconazole. He continued to improve, and his inflammatory markers normalised after 8 weeks of treatment. Prednisolone was started for COVID-19 related pneumonitis. Long-term antifungal treatment is advisable, and we aim to complete 12 weeks of treatment.

**Case report - Key learning points**
 

1. Patients with SARS-CoV-2 infection, particularly those requiring ICU admission were at risk of developing superinfections with multidrug-resistant Gram-negative bacteria or fungal infections.
2. Candida albicans sacroiliitis is rare therefore early aspiration/biopsy is essential for the management.
3. Longer treatment is needed in osteoarticular candida infections, even up to 6 or 12 months, therefor long-term close monitoring of this patients is essential.
4. The utility and timing of reimaging patients following such infections is still unclear
5. Close multidisciplinary and interdisciplinary team collaboration is essential in the management of this complex patients

